# Investigation of the preservation effect of canagliflozin on pancreatic beta cell mass using SPECT/CT imaging with ^111^In-labeled exendin-4

**DOI:** 10.1038/s41598-019-54722-w

**Published:** 2019-12-04

**Authors:** Keita Hamamatsu, Hiroyuki Fujimoto, Naotaka Fujita, Takaaki Murakami, Masaharu Shiotani, Kentaro Toyoda, Nobuya Inagaki

**Affiliations:** 1 Department of Diabetes, Endocrinology and Nutrition, Graduate School of Medicine, Kyoto University, 54 Shogoin Kawahara-cho, Sakyo-ku, Kyoto 606-8507 Japan; 2 Radioisotope Research Center, Agency of Health, Safety and Environment, Kyoto University, Yoshida Konoe-cho, Sakyo-ku, Kyoto 606-8501 Japan; 3 Sohyaku, Innovative Research division, Mitsubishi Tanabe Pharma Corporation, 2-2-50 Kawagishi, Toda, Saitama 335-8505 Japan

**Keywords:** Molecular imaging, Type 2 diabetes

## Abstract

Radiolabeled exendin derivatives are promising for non-invasive quantification of pancreatic beta cell mass (BCM); longitudinal observation of BCM for evaluation of therapeutic effects has not been achieved. The aim of this study is to demonstrate the usefulness of our developing method using [Lys^12^(^111^In-BnDTPA-Ahx)]exendin-4 to detect longitudinal changes in BCM. We performed a longitudinal study with obese type 2 diabetes model (db/db) mice administered canagliflozin, which is reported to preserve BCM. Six-week-old mice were assigned to a canagliflozin-administered group or a control group. Blood glucose levels of the canagliflozin group were significantly lower than those of the control group. Plasma insulin levels, insulin secretion during OGTT and insulin content in the pancreas were preserved in the canagliflozin group in comparison with those in the control group. According to SPECT/CT imaging analysis using [Lys^12^(^111^In-BnDTPA-Ahx)]exendin-4, pancreatic uptake was significantly decreased in the control group, whereas there was no significant change in the canagliflozin group. After nine weeks, both pancreatic uptake and BCM of the canagliflozin group were significantly higher than those of the control group, and a correlation between them was observed. In conclusion, our imaging method confirmed the BCM-preservation effect of canagliflozin, and demonstrated its potential for longitudinal evaluation of BCM.

## Introduction

Impaired insulin secretion is an important pathophysiological factor in the development of type 2 diabetes. This may implicate dysfunction of individual beta cells and a decrease of beta cell mass (BCM). It was shown in animal models of type 2 diabetes that chronic hyperglycemia induces beta cell apoptosis^1,2^ and triggers loss of beta cell differentiation^3^. Butler *et al*. showed by using autopsy data that BCM is decreased in patients with impaired fasting glucose or type 2 diabetes^4^.

Non-invasive methods to quantify BCM were unavailable until recently, and it has not been possible to observe longitudinal changes in BCM in same subject. It has therefore been impossible to evaluate effects of treatment by anti-diabetic agents on BCM in detail over time.

We have been developing imaging methods using radiolabeled exendin derivatives targeting the glucagon-like peptide-1 (GLP-1) receptor, with the aim of quantifying BCM by measuring probe accumulation in the pancreas^5–7^. We developed [Lys^12^(^111^In-BnDTPA-Ahx)]exendin-4 ([^111^In]Ex4), and reported specific binding of this probe to the GLP-1 receptor^8^. We also established an analytical method for single photon emission computed tomography (SPECT) imaging of living mice to quantify pancreatic uptake of [^111^In]Ex4^9^, and reported that pancreatic uptake is correlated with BCM as calculated by the product of the pancreatic weight and the area ratio of tissue immunostained with anti-insulin antibody to whole pancreas^10^. *In vivo* SPECT imaging of a living animal is non-invasive and repeatable; our imaging method can thus realize longitudinal observation of BCM and become a useful tool for basic research on the pathophysiology of diabetes and the development of drugs for the treatment of BCM.

Recently, administration of sodium glucose transporter-2 inhibitors (SGLT2i) to obese type 2 diabetes model mice such as db/db mice was shown to preserve BCM^11,12^. However, in these studies, longitudinal changes of BCM in the same individual could not be discerned because the mice were sacrificed at the respective time points for evaluation of BCM using the harvested pancreata. In the current study, to demonstrate that our SPECT/CT imaging of pancreatic beta cells with [^111^In]Ex4 is useful for longitudinal observation of BCM, we performed a longitudinal study on BCM of db/db mice administered canagliflozin.

## Materials and Methods

### Animals

Five-week-old male BKS.Cg- + Lepr^db^/ + Lepr^db^/Jcl^*^ (db/db) mice and BKS.Cg-m + / + Lepr^db^/Jcl^*^ (db/m) mice, the lean control of db/db mice, were purchased from CLEA Japan (Tokyo, Japan). At six weeks of age, db/db mice were assigned to the db/db(c+) or db/db(c−) groups so as not to create a significant difference in body weight or random blood glucose and plasma insulin levels; all db/m mice were assigned to group db/m(c−). Mice in group db/db(c+) were fed normal laboratory chow CE-2 (CLEA Japan) with canagliflozin as a food admixture (0.005%, w/w) after six weeks of age; mice in group db/db(c−) and db/m(c−) were fed CE-2 without canagliflozin. Canagliflozin was provided by Mitsubishi Tanabe Pharma Corporation.

Animal care and experimental procedures were approved by the Kyoto University Animal Care Committee and were performed in accordance with the institutional guidelines of Kyoto University.

### Measurements of body weight and weekly food consumption, random blood glucose and plasma insulin levels

Body weight and weekly food consumption, random blood glucose and plasma insulin levels were measured under free feeding every week from 6 to 15 weeks of age. Using 50 µl blood samples collected from the tail vein, the blood glucose levels were monitored with PocketChem BG PG-7320 (ARKRAY, Inc., Kyoto, Japan), and the plasma insulin levels were measured with Ultra Sensitive Mouse Insulin ELISA Kit (Morinaga Institute of Biological Science, Inc., Yokohama, Japan).

### Radiolabeling of [Lys^12^(BnDTPA-Ahx)]exendin-4 with ^111^Indium

The precursor peptide [Lys^12^(BnDTPA-Ahx)]exendin-4 was synthesized by KNC Laboratories (Kobe, Japan). ^111^InCl_3_ was purchased from Nihon Medi-Physics (Tokyo, Japan). Radiolabeling of [Lys^12^(BnDTPA-Ahx)]exendin-4 with ^111^In was performed as we previously reported^8^. In brief, a 0.01 μM [Lys^12^(BnDTPA-Ahx)]exendin-4 precursor solution was prepared in a 0.01 M MES buffer (pH 5.5) containing 0.1% Tween-80 as a solubilizer. Ten mL of 0.01 µM precursor and 1 mL of 74 MBq ^111^InCl_3_ solution were mixed and incubated for 15 min at room temperature, and subsequently applied to a Sep-Pak Light C18 Cartridge (Waters, Milford, MA, USA). The cartridge was eluted with ethanol; the eluent was concentrated using argon gas flow and diluted with normal saline so that the specific radioactivity would be 30 MBq/mL at the time of injection.

### SPECT/CT scans and image analysis

SPECT/CT scans of mice were performed every three weeks with a Triumph LabPET12/SPECT4/CT (TriFoil Imaging Inc., Chatsworth, CA, USA). All mice were under isoflurane anesthesia during SPECT/CT scans starting from 30 min after intravenous injection of 3.0 MBq [^111^In]Ex4.

SPECT scans were performed using multi-pinhole collimators (1.0 mm diameter). Projection data were acquired using an energy window between 150 and 192 keV over 360° in 32 projections of 60 s each, and reconstructed using a 3-dimensional ordered-subsets expectation maximization algorithm. Subsequently, computed tomography (CT) images were acquired at 60 kV and 310 mA, and were reconstructed using a modified 3D cone-beam Feldkamp algorithm. This instrument does not have the function of attenuation correction.

Using Amira software version 5.6.0 (FEI Visualization Sciences Group, Düsseldorf, Germany), the uptake values of the pancreas were measured from the SPECT/CT image, as we previously reported^9^. In brief, regions of interest (ROIs) of the pancreas were selected to have volume no less than 40% of the whole pancreas and to eliminate the influence of high renal uptake caused by renal excretion, and the uptake values (counts/mm^3^) of the pancreatic ROIs were measured with software.

The uptake values were converted to radioactivities (Bq/g) with a conversion factor derived from previous *ex vivo* study^9^. The percentage of pancreatic radioactivities in injected dose per gram of pancreatic tissue (%ID/g) was calculated by dividing the pancreatic radioactivity (Bq/g) by the injected dose of [^111^In]Ex4 (Bq).

### Immunohistochemical quantification of BCM

At 15 weeks of age, pancreata were resected from mice observed with SPECT/CT, fixed in 10% formalin and embedded in paraffin. Ten sections of 5 μm thickness no less than 100 μm apart from each other were immunostained with rabbit polyclonal anti-insulin antibody (sb9168; Santa Cruz Biotechnology, Santa Cruz, CA, USA) and mouse monoclonal anti-glucagon antibody (ab10988; Abcam plc, Cambridge, UK) used as primary antibodies; Alexa Fluor 488 goat anti-rabbit IgG and Alexa Fluor 546 goat anti-mouse IgG (A-11034 and A-11030; Thermo Fisher Scientific, Waltham, MA, USA) were used as secondary antibodies, as previously reported^10^.

BCM was measured by the same method as in previous reports^10,13–15^ as outlined following. The insulin-positive area was determined by ROIs over pancreatic tissue immunostained with anti-insulin antibody. Adjacent sections were stained with hematoxylin and eosin, and then were used to determine total pancreatic area. The insulin-positive ratio was calculated by dividing the insulin-positive area by the total pancreatic area. BCM was calculated as the product of the weight of the resected pancreas and the insulin-positive ratio.

### Oral glucose tolerance test (OGTT)

OGTT was performed every three weeks from 6 to 15 weeks of age in animals not observed with SPECT/CT. At the respective ages, 2 g/kg body weight glucose solution was orally administered to mice in each group after overnight fasting. Blood samples were collected from the tail vein before and 15, 30, 60, 90 and 120 min after loading glucose. Blood glucose levels were measured with Glucose CII Test Wako Kit (FUJIFILM Wako Pure Chemical Corporation, Osaka, Japan), as plasma glucose levels after glucose loading exceeded the upper reference limit of PocketChem in some mice. Plasma insulin levels were measured with the same method as weekly measurement.

### Measurement of insulin content in pancreas

Insulin content in the pancreas was examined every three weeks from 6 to 15 weeks of age in animals not observed with SPECT/CT or OGTT. At the respective ages, the mice were sacrificed, and the harvested pancreata were homogenized using ultrasonic waves generated by Handy Sonic UR-20P (TOMY SEIKO CO.,LTD., Tokyo, Japan) in 1.0 mL acid-ethanol (0.18 M HCl in 75% ethanol) and incubated at 4 °C overnight. Insulin content in the pancreas (µg/g pancreatic weight) was calculated as insulin content measured with the aforementioned ELISA kit divided by pancreatic weight.

### Statistical analysis

Values are expressed as mean ± standard deviation (SD). Statistical analysis was performed using IBM SPSS Statistics for Windows (Version 22.0, IBM Corp. Armonk, NY, USA). One-way analysis of variance and Tukey test were used to compare continuous variables among three groups. One-way repeated measures analysis of variance and Fisher’s least significant difference methods were used to detect significant changes over time in each group. In case of comparison of data from OGTT and insulin content at six weeks of age between only two groups due to lack of the db/db(c+) group, we used unpaired two-tailed *t* tests. Correlations were determined using Pearson’s test. The level of significance was set at *p* < 0.05.

## Results

### Body weight and food consumption, random blood glucose and plasma insulin levels

**Figure 1:**
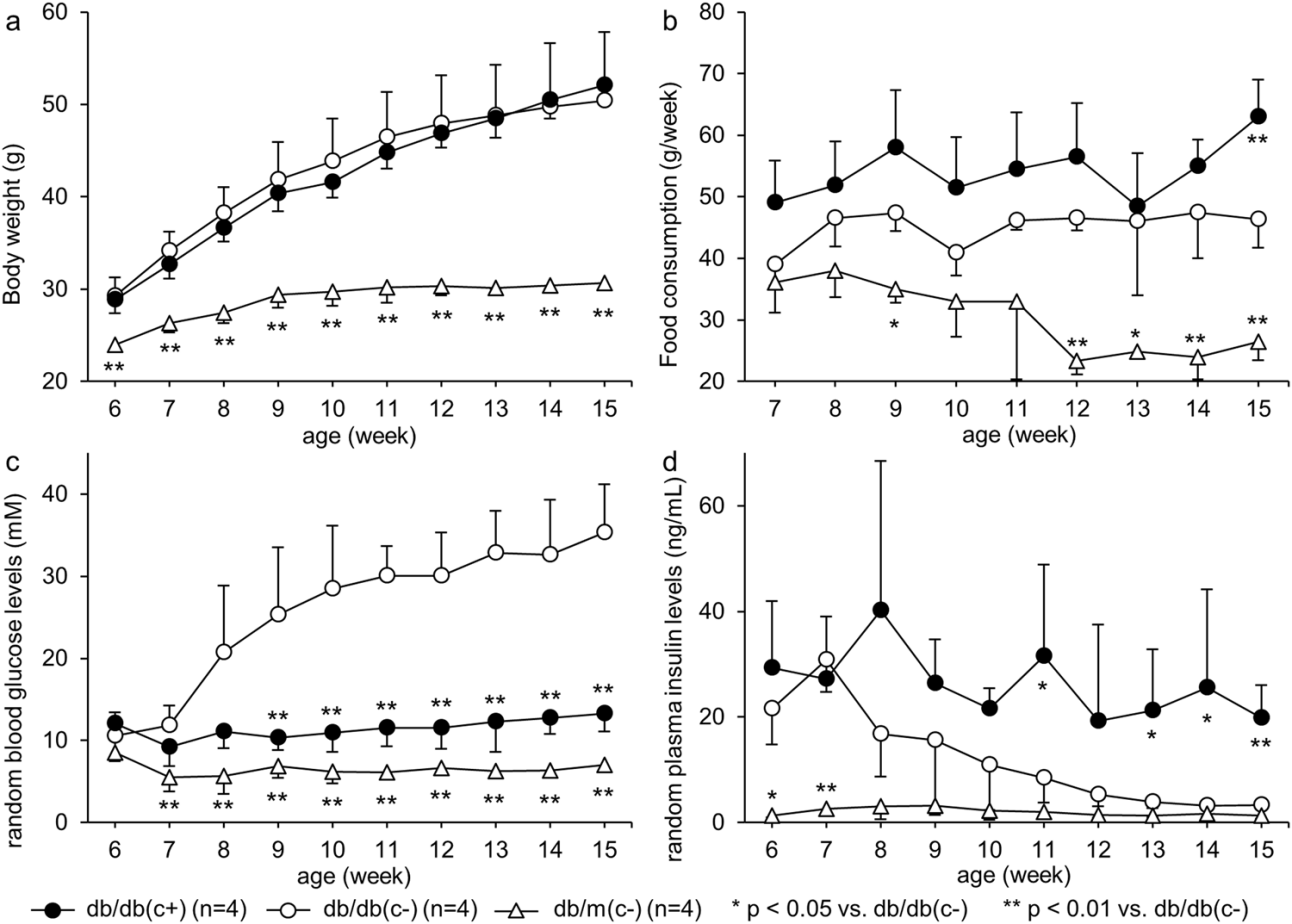
Body weight, food consumption, random plasma glucose and insulin levels. (**a**) Body weight and (**b**) weekly food consumption, (**c**) random blood glucose and (**d**) plasma insulin levels were measured under free feeding every week from 6 to 15 weeks of age. During the study, there was no significant difference in body weight between group db/db(c+) and db/db(c−). There were significant differences in food consumption, blood glucose and plasma insulin levels between the two groups.

Weekly body weight and food consumption, random blood glucose and plasma insulin levels are shown in Fig. 1. Body weights were increased in both group db/db(c+) and db/db(c−). In the early phase of the observation period, the body weight of group db/db(c+) tended to be less than that of group db/db(c−); on the other hand, in the late phase, the weight gain of group db/db(c+) tended to be greater than that of group db/db(c−), but there was no significant difference in body weight between the two groups during the study. Body weight of group db/m(c−) was significantly lower than that of group db/db(c−) throughout the observation period. Food consumption of group db/db(c+) tended to be greater than that of group db/db(c−), but there was no significant difference between the two groups except at 15 weeks of age. Food consumption in group db/m(c−) was significantly less than that in group db/db(c−) at 9 and 12–15 weeks of age. There was no significant difference in blood glucose levels among the three groups at 6 weeks of age. After 9 weeks of age, the blood glucose levels of group db/db(c+) were significantly lower than that of group db/db(c−). The blood glucose levels of group db/m(c−) were significantly lower than those of group db/db(c−) after 7 weeks of age. The blood glucose levels of group db/m(c−) tended to be lower than those of group db/db(c+), but there was not a significant difference except at 11 weeks of age (*p* = 0.031). The plasma insulin levels of group db/db(c−) were significantly decreased, whereas those of group db/db(c+) were preserved; significant differences between the two groups were observed at 11 and 13–15 weeks of age. The plasma insulin levels of group db/m(c−) were significantly lower than those of group db/db(c−) at 6 weeks of age, but this difference was eliminated after 9 weeks of age.

### Blood glucose and plasma insulin levels during OGTT

**Figure 2:**
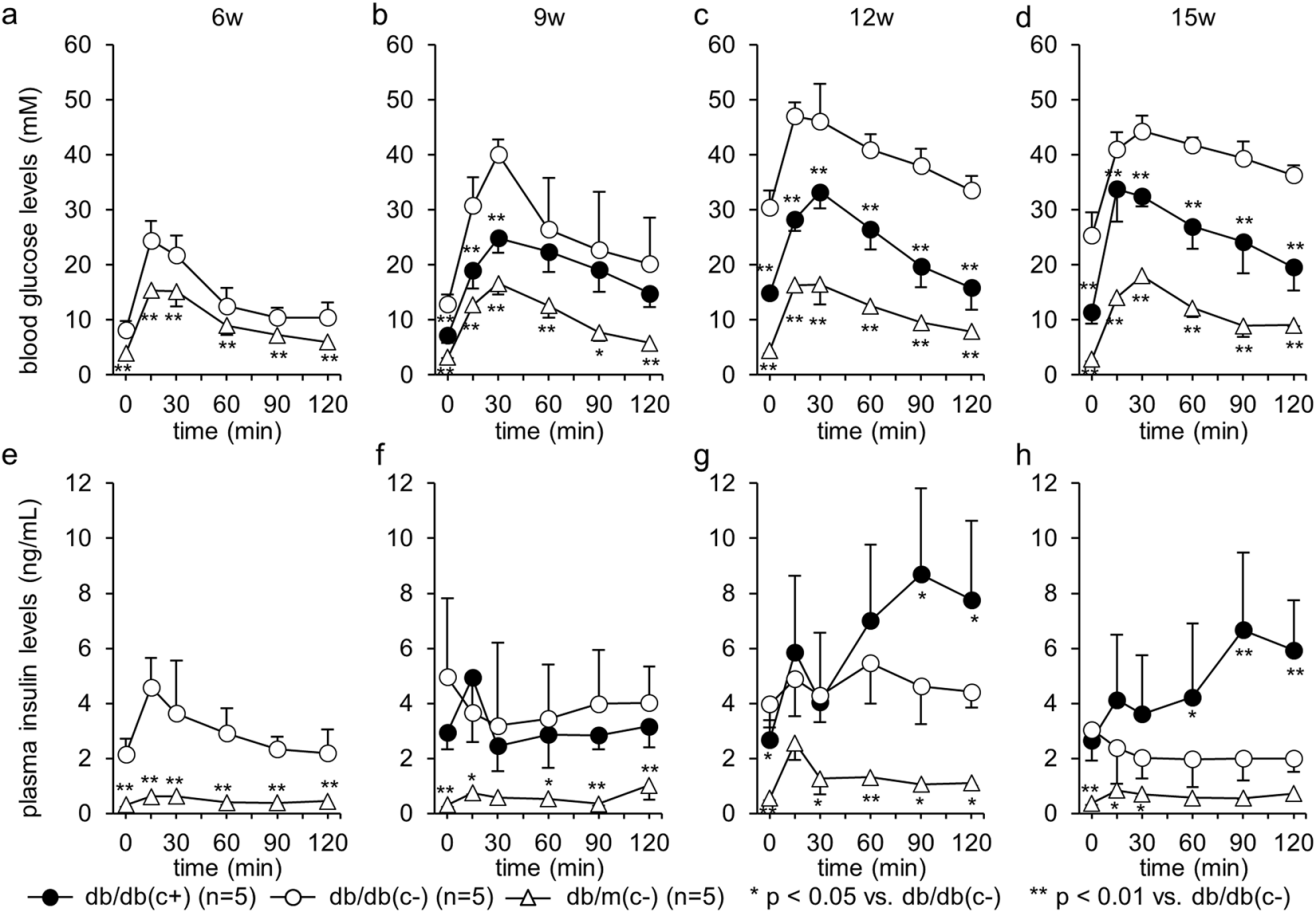
Plasma glucose and insulin levels during OGTT. OGTT was performed every three weeks from 6 to 15 weeks of age using 2 g/kg body weight glucose. After 9 weeks of age, the plasma glucose levels in group db/db(c+) were significantly lower than those in group db/db(c−). Initial insulin secretion was impaired in group db/db(c−); on the other hand, in db/db(c+), initial insulin secretion was preserved, and plasma insulin levels at 90 and 120 min were significantly higher than those in group db/db(c−) after 12 weeks of age.

After 9 weeks of age, the plasma glucose levels in group db/db(c+) were significantly lower than those in group db/db(c−). In group db/db(c−), blood glucose levels at both 120 min and peak were significantly higher after 9 weeks of age than those at 6 weeks of age. Even in group db/db(c+), blood glucose levels at 120 min and peak were significantly higher after 9 or 12 weeks of age than those at 6 weeks of age before administration of canagliflozin, whereas the blood glucose levels at 120 min were <11.1 mM. There was no significant change in peak glucose levels in group db/m(c−) during the study. (Fig. 2a–d)

Initial insulin secretion, which is defined as the subtraction of the insulin level at 0 min from that at 15 min, at six weeks of age was compared to that at later weeks of age in each group. Initial insulin secretion at 9 and 15 weeks of age was significantly lower than that at 6 weeks of age in group db/db(c−) (2.43 ± 1.30, −1.30 ± 2.19, 0.91 ± 1.32 and −0.14 ± 0.95 at 6, 9, 12 and 15 weeks of age, respectively; *p* = 0.001, 0.055 and <0.001 at 9, 12 and 15 vs. 6 weeks of age, respectively). On the other hand, in group db/db(c+), no significant difference in these values was observed (2.43 ± 1.30, 1.98 ± 2.03, 3.18 ± 2.46 and 1.00 ± 1.70 at 6, 9, 12 and 15 weeks of age, respectively). After 12 weeks of age, plasma insulin levels at 90 and 120 min in group db/db(c+) were significantly higher than those in group db/db(c−). (Fig. 2e–h)

### Insulin content in pancreas

**Figure 3:**
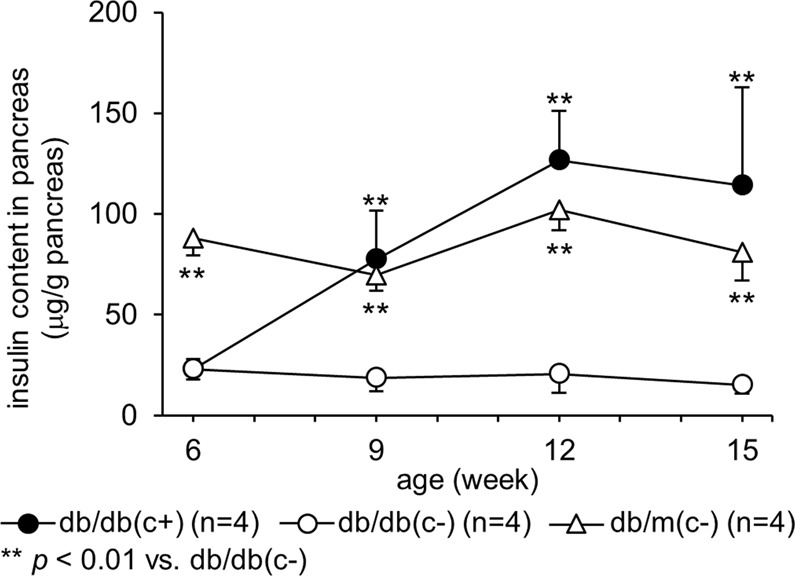
Insulin content in pancreas. Insulin contents in the pancreas were examined every three weeks from 6 to 15 weeks of age. Insulin content in group db/db(c+) was significantly higher than that of group db/db(c−) at 9 weeks of age or later. A significant difference between insulin content of group db/db(c−) and that of group db/m(c−) was observed at respective ages.

The insulin content of group db/db(c+) was significantly higher than that of group db/db(c−) at 9 weeks of age and later. A significant difference between insulin content of group db/db(c−) and that of group db/m(c−) was observed at the respective ages. (Fig. 3).

### Immunohistochemical study with pancreas resected at 15 weeks of age

**Figure 4:**
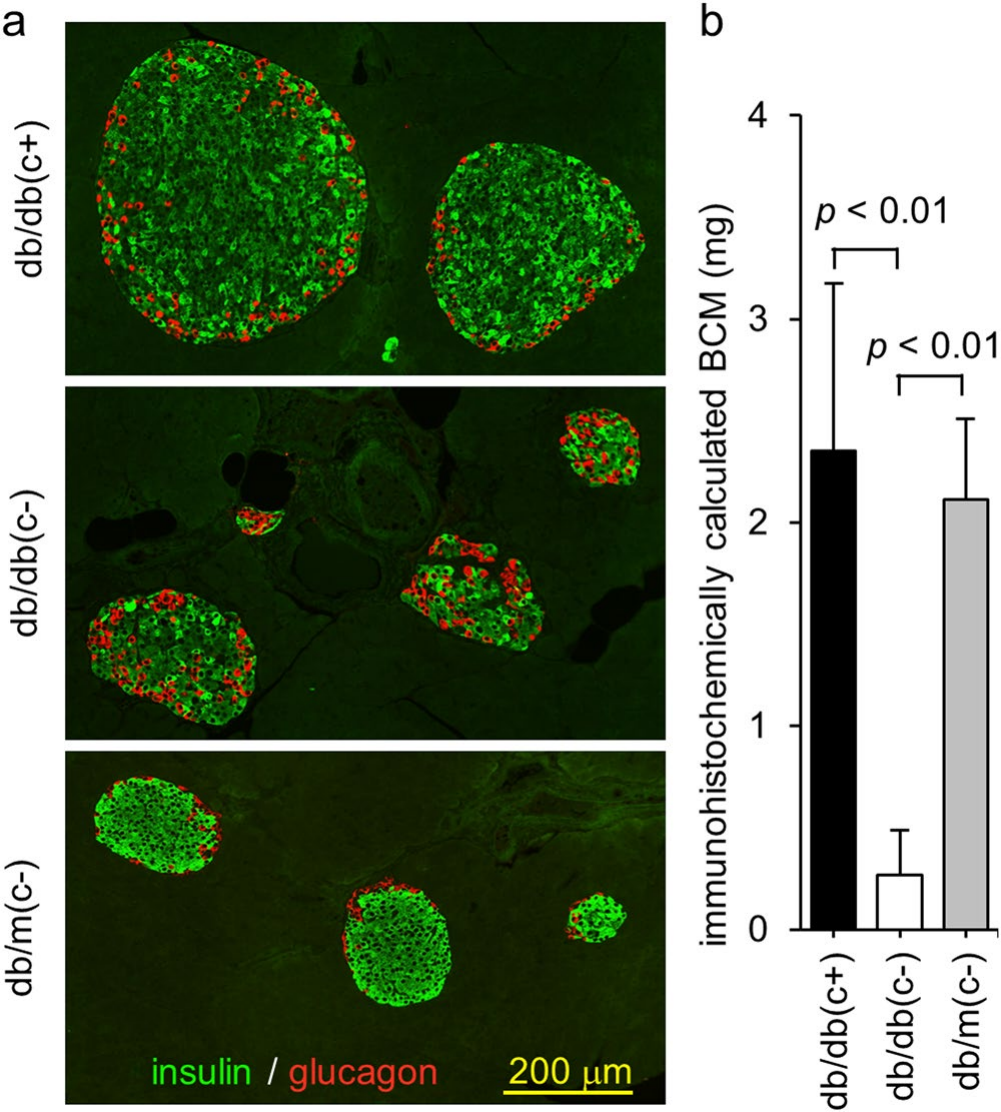
Immunohistochemical study of pancreas resected at 15 weeks of age. At 15 weeks of age, pancreata were resected and pancreatic sections were stained with anti-insulin and anti-glucagon antibodies. (**a**) Enlargement of pancreatic islets in group db/db(c+) and disturbance of localization of alpha and beta cells in group db/db(c−) were observed. (**b**) BCM was calculated as the product of the insulin positive ratio and pancreatic weight. BCM in group db/db(c+) was significantly greater than that in group db/db(c−), and was equivalent to that in group db/m(c−).

Pancreatic alpha and beta cells in mice are known to locate in the peripheral and central area of islets, respectively. Immunohistochemical examination showed that this localization was applicable to group db/m(c−) and db/db(c+); however, in group db/db(c−), this localization was disturbed. Enlargement of pancreatic islets also was observed in group db/db(c+) (Fig. 4a). The BCM of group db/db(c+) was significantly greater than that of group db/db(c−), and was equivalent to that of group db/m(c−) (Fig. 4b).

### SPECT/CT image and pancreatic uptake

**Figure 5:**
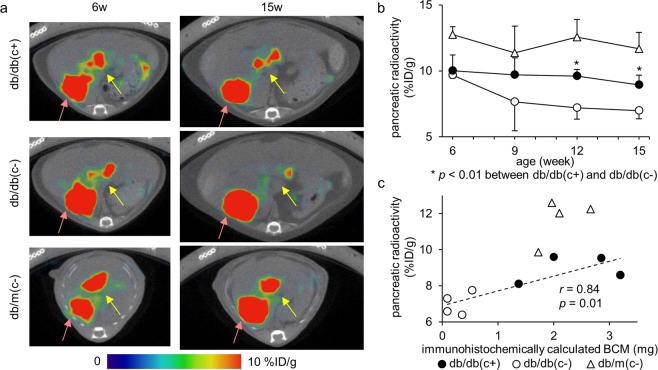
SPECT/CT images and pancreatic uptake. SPECT/CT scans were performed every three weeks from 6 to 15 weeks of age 30 min after intravenous injection of 3.0 MBq [^111^In]Ex4. (**a**) Axial images are shown. Yellow arrows show pancreatic uptake and red arrows show right renal uptake. Sagittal and coronal images can be found as Supplementary Figure S1. (**b**) The pancreatic radioactivity (%ID/g) was significantly decreased in group db/db(c−) after 12 weeks of age, whereas no significant change was observed in group db/db(c+). After 12 weeks of age, the pancreatic radioactivity of group db/db(c+) was significantly higher than that of group db/db(c−). A plot of individual mice can be found as Supplementary Figure S2. (**c**) Correlation between the pancreatic radioactivity and immunohistochemically calculated BCM was observed in group db/db(c+) and db/db(c−). However, the pancreatic radioactivity of group db/m(c−) could not be plotted in the same straight line as that in group db/db(c+) and db/db(c−).

Typical fused images of [^111^In]Ex4 SPECT and CT of mice in the respective groups are shown in Fig. 5a. The pancreatic radioactivity (%ID/g) was significantly decreased in group db/db(c−) after 12 weeks of age; on the other hand, no significant change was observed in group db/db(c+). After 12 weeks of age, pancreatic radioactivity of group db/db(c+) was significantly higher than that of group db/db(c−) (Fig. 5a,b). A correlation between the pancreatic radioactivity and BCM was observed in group db/db(c+) and db/db(c−) (*r* = 0.84). However, the pancreatic radioactivity of group db/m(c−) did not plot in the same straight line as that of group db/db(c+) and db/db(c−) (Fig. 5c).

## Discussion

Radiolabeled exendin derivatives are promising probes for non-invasive quantification of BCM. Some groups, including ours, are developing radiolabeled exendin derivatives^5–8,16,17^. However, most of the previous reports have used SPECT scan not with living mice but with resected pancreata^18,19^, and longitudinal observation of BCM has not been achieved. In order to enable longitudinal observation, we established an analytical method using SPECT imaging of living mice to quantify pancreatic uptake of [^111^In]Ex4^9^; we reported a correlation between pancreatic uptake and BCM and also a longitudinal decrease in BCM in NOD mice, an animal model of type 1 diabetes^10^. As the next step, in the present study, we were able to follow longitudinal changes in BCM of type 2 diabetic model mice treated with anti-diabetic agents for evaluation of the therapeutic effect.

We used SPECT imaging even though PET is superior to SPECT for quantitative evaluation in general. Indeed, ^68^Ga- or ^18^F- labeled exendin for PET imaging has been reported^20,21^. However, due to their short half-life, basic research requiring a certain number of scans for every probe synthesis such as the current study needs further investigation to confirm imaging conditions. There are only a few reports on ^64^Cu- or ^89^Zr- labeled exendin for PET imaging, which have a longer half-life^20,22^, and longitudinal study using these probes is worth considering in the future. In this study, we used ^111^In-labeled exendin for SPECT imaging for its availability and usefulness, which we have previously shown in small animal study^8–10^.

We carefully determined which agent was most suited for the current study. Dipeptidyl peptidase-4 inhibitor (DPP-4i) and GLP-1 analogue are known to preserve BCM^23,24^, but these incretin-based drugs might well affect the biodistribution of radiolabeled exendin. SGLT2i does not act directly on pancreatic beta cells, but some groups have reported that administration of SGLT2i to db/db mice was able to preserve BCM^11,12^. Therefore, this agent was chosen for the current trial with our imaging method.

In this study, we observed no significant difference in body weight between group db/db(c+) and db/db(c−), although it is known from clinical study that canagliflozin reduces body weight of patients with type 2 diabetes^25^. Some studies have shown that body weight of db/db mice administered SGLT2i was greater than that of untreated mice, which was considered to be due to decreased insulin secretion leading to lack of weight gain in the untreated group, and, conversely, preserved insulin secretion and hyperphagia in the SGLT2i-administered group. In the current study, food intake of group db/db(c+) was greater than that of group db/db(c−), and decreased insulin secretion in group db/db(c−) and preserved insulin secretion in group db/db(c+) were shown by weekly blood collection or OGTT. These results are comparable with previous reports^26,27^.

We found a correlation between BCM and pancreatic uptake as calculated from the SPECT/CT images of mice, using repeated administration of the probe four times. In a previous report, our findings were limited to a first or second injection; we have now shown that our non-invasive method can accurately quantify longitudinal change of BCM in the same animal by using four repetitions. Longitudinal change of BCM could be estimated accurately in the same individual by comparing four SPECT/CT images. We were thus able to estimate the treatment effect of canagliflozin on BCM.

There are some limitations to our method. First, simple comparison of different strains using our imaging method may not be appropriate. In this study, pancreatic uptake of group db/m(c−) was significantly higher than that of group db/db(c+), although there was no significant difference between group db/db(c+) and db/m(c−) regarding immunohistochemically calculated BCM. For this reason, the pancreatic radioactivity of db/m mice did not plot in the same straight line as that of db/db mice. This phenomenon may also be partly explained by the strain difference, as pancreatic uptake of ^111^In-labeled exendin has been reported to vary by the strain of mice^28^. In addition, a defect in the immunohistochemical method for calculation of BCM using anti-insulin antibody, which was the sole method used to estimate BCM, may also be partly responsible for the phenomenon. Immunohistochemistry with the antibody used does not stain beta cells uniformly; the average of fluorescent intensity in the insulin positive area tended to be higher in group db/m(c−) than that in group db/db(c+) in this study. Because the same threshold of fluorescence intensity was adopted for fair comparison when selecting ROI of the insulin positive areas, the BCM of db/m mice might thus be overestimated. Besides this point, there is another advantage to our imaging study: the observable volume of the pancreas was higher than that in the immunohistological study, which was performed in only a part of the pancreas.

Second, pancreatic uptake derived from SPECT/CT images shows BCM per unit volume, not BCM itself. At 15 weeks of age, the product of pancreatic uptake and the volume of harvested pancreas were used for comparison to immunohistochemical BCM. However, at other ages during the study, pancreatic volume could not be measured. For that reason, when pancreatic volume changes largely due to growth or atrophy, strict estimation of BCM change is difficult. This problem occurs in small animals such as mice because the pancreas cannot be delineated at each time point in reference to the CT image. In our experimental conditions, pancreatic weights did not significantly change from 6 to 15 weeks of age in the respective groups, and there was no significant difference in pancreatic weights between group db/db(c+) and db/db(c−) at respective ages. Thus, this problem does not alter the interpretation of results.

In the current study, in spite of the 87.7% lower BCM of group db/db(−) compared to that of group db/db(+) (Fig. 4b), the radioactivity (%ID/g) of group db/db(−) was 21.9% lower than that of group db/db(+) (Fig. 5b). These results are comparable with previous reports showing no more than a 40% decrease of pancreatic uptake in mice in which the beta cells were destroyed. This can be explained by the fact that ^111^In-labeled exendin accumulates not only to beta cells but also to exocrine pancreas^10,29,30^ due to the much greater volume of exocrine pancreas compared to that of beta cells even though the GLP-1 receptor expression level in exocrine pancreas is much less than that in beta cells^28^.

Regardless of the above limitations, which require further investigation, longitudinal observation of BCM with the present imaging method is novel, and represents a breakthrough in basic research on the pathophysiology of diabetes that will be a useful tool for the non-clinical studies aiming for the development of drugs to enlarge BCM.

In conclusion, a longitudinal observation method using imaging with [^111^In]Ex4 shows that BCM of db/db mice treated with canagliflozin was significantly preserved compared to that of non-treated db/db mice. Thus, our imaging method can estimate the longitudinal change of BCM within the same individual.

## Supplementary information

Supplementary Figure S1 and S2

## Data Availability

The data that support the findings of this study are available from the corresponding author upon reasonable request.
