# Supplementary material for: Investigation of the preservation effect of canagliflozin on pancreatic beta cell mass using SPECT/CT imaging with 111In-labeled exendin-4
Source: Sci Rep. 2019 Dec 4;9:18338. doi: 10.1038/s41598-019-54722-w (PMC6893013; doi:10.1038/s41598-019-54722-w)
Supplement: Supplementary file 1 — Supplementary Figure S1 and S2 [file 41598_2019_54722_MOESM1_ESM.docx]

**Supplemental Materials**

Investigation of the preservation effect of canagliflozin on pancreatic beta cell mass using SPECT/CT imaging with ^111^In-labeled exendin-4.

Keita Hamamatsu, Hiroyuki Fujimoto, Naotaka Fujita, Takaaki Murakami, Masaharu Shiotani, Kentaro Toyoda, Nobuya Inagaki

**Supplementary Figure S1. SPECT/CT images.**

SPECT/CT scans were performed every three weeks from 6 to 15 weeks of age 30 min after intravenous injection of 3.0 MBq [^111^In]Ex4. Yellow arrows show pancreatic uptake and red arrows show right renal uptake. (a) Sagittal and (b) coronal images are shown.

**
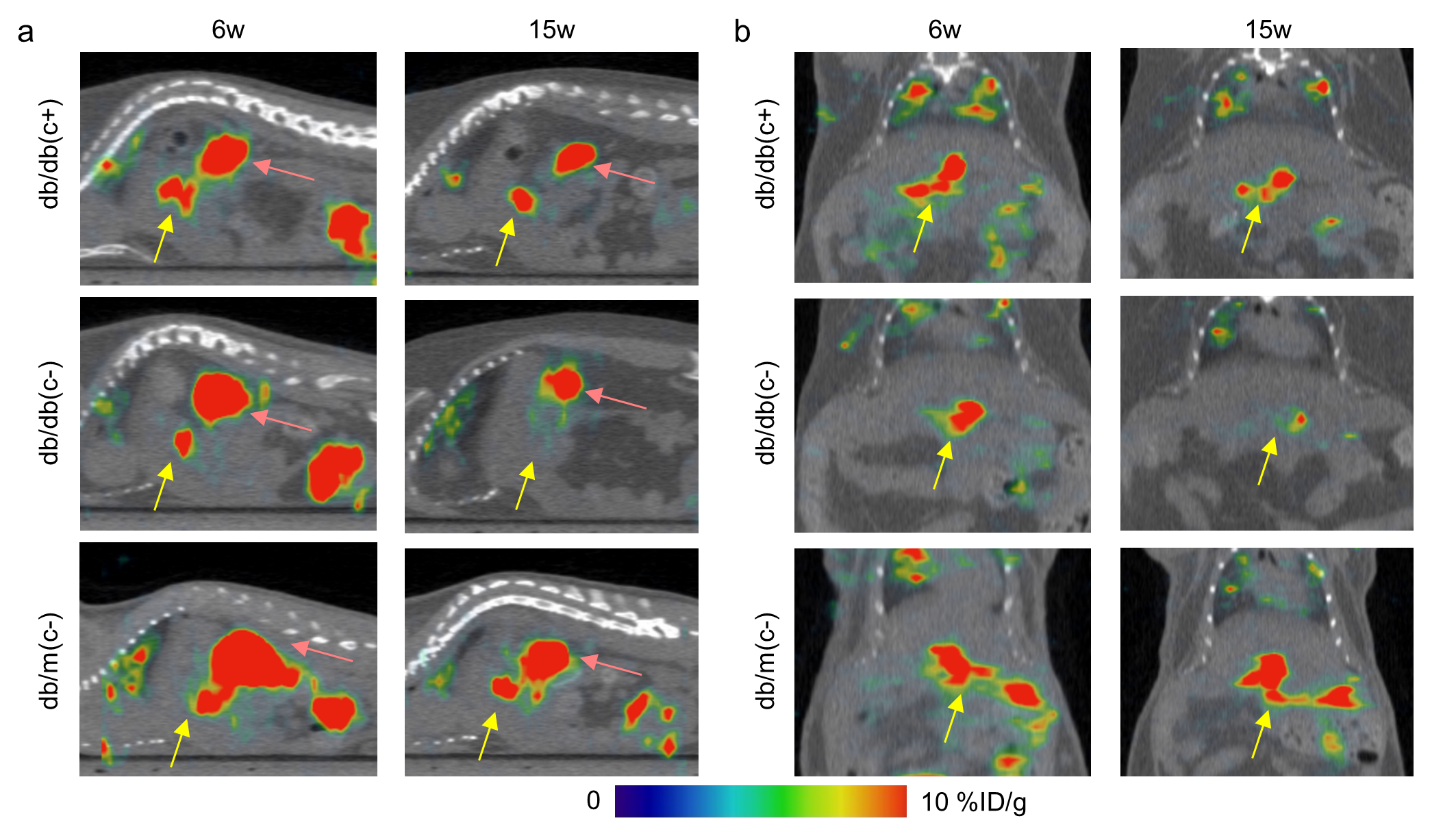
**

**Supplementary Figure S2. Transition of the pancreatic radioactivity of individual mice.**

SPECT/CT scans were performed every three weeks from 6 to 15 weeks of age 30 min after intravenous injection of 3.0 MBq [^111^In]Ex4. The pancreatic radioactivity (%ID/g) was decreased in group db/db(c-), whereas no significant change was observed in group db/db(c+).

**
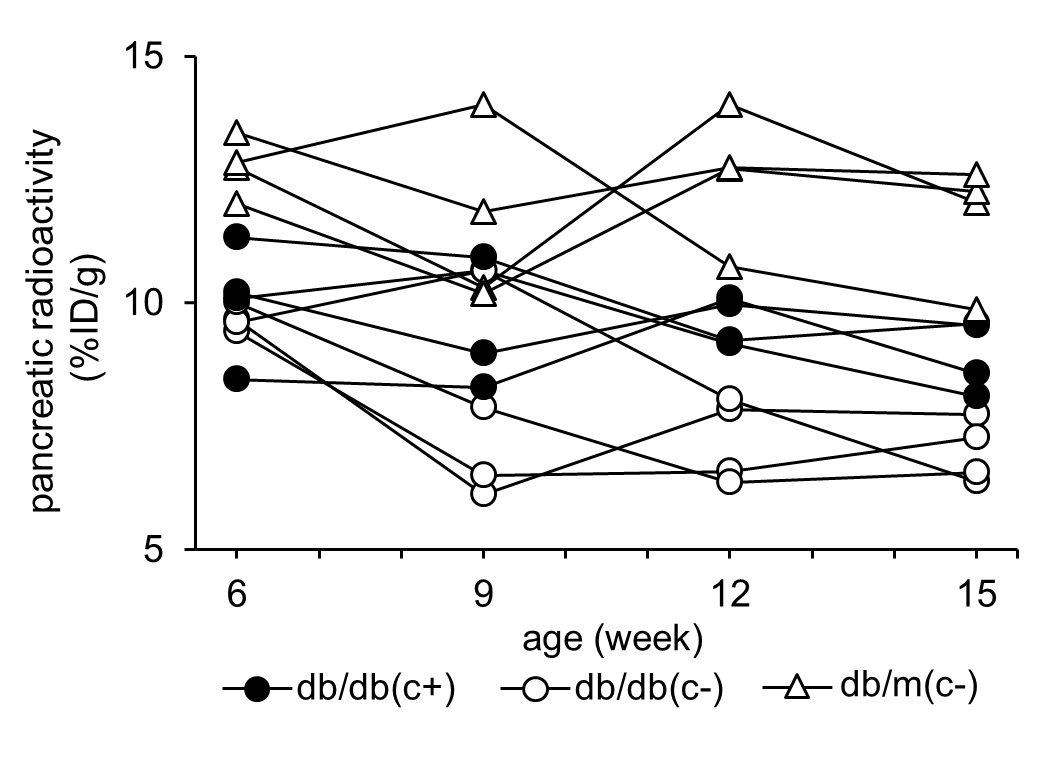
**
